# Differences in enteric neuronal density in the NSE-Noggin mouse model across institutes

**DOI:** 10.1038/s41598-024-54337-w

**Published:** 2024-02-14

**Authors:** Simone L. Schonkeren, Meike S. Thijssen, Musa Idris, Kim Wouters, Joëlle de Vaan, Andreas Teubner, Marion J. Gijbels, Werend Boesmans, Veerle Melotte

**Affiliations:** 1 Department of Pathology, GROW–Research Institute for Oncology and Reproduction, Maastricht University Medical Center, Maastricht, The Netherlands; 2 Biomedical Research Institute (BIOMED), Hasselt University, Hasselt, Belgium; 3 Department of Clinical Genetics, Erasmus University Medical Center, Rotterdam, The Netherlands; 4 Central Animal Facility, Faculty of Health, Medicine & Life Sciences, Maastricht University, Maastricht, The Netherlands; 5 Department of Medical Biochemistry, Amsterdam Cardiovascular Sciences: Atherosclerosis & Ischemic Syndrome, Amsterdam Infection and Immunity: Inflammatory Diseases, Amsterdam UMC location University of Amsterdam, Amsterdam, The Netherlands

**Keywords:** Experimental organisms, Peripheral nervous system, Gastrointestinal models

## Abstract

The enteric nervous system (ENS) is a large and complex part of the peripheral nervous system, and it is vital for gut homeostasis. To study the ENS, different hyper- and hypo-innervated model systems have been developed. The *NSE-Noggin* mouse model was described as one of the few models with a higher enteric neuronal density in the colon. However, in our hands *NSE-Noggin* mice did not present with a hyperganglionic phenotype. *NSE-Noggin* mice were phenotyped based on fur appearance, genotyped and DNA sequenced to demonstrate transgene and intact NSE-Noggin-IRES-EGFP construct presence, and RNA expression of *Noggin* was shown to be upregulated. Positive EGFP staining in the plexus of *NSE-Noggin* mice also confirmed Noggin protein expression. Myenteric plexus preparations of the colon were examined to quantify both the overall density of enteric neurons and the proportions of enteric neurons expressing specific subtype markers. The total number of enteric neurons in the colonic myenteric plexus of transgenic mice did not differ significantly from wild types, nor did the proportion of calbindin, calretinin, or serotonin immunoreactive myenteric neurons. Possible reasons as to why the hyperinnervated phenotype could not be observed in contrast with original studies using this mouse model are discussed, including study design, influence of microbiota, and other environmental variables.

## Introduction

The enteric nervous system (ENS) consists of neurons and glia structured in a ganglionic network along the length of the gut. It regulates vital gastrointestinal processes such as motility, fluid exchange, blood flow, and immune responses^1^. Many diseases are caused or influenced by ENS dysfunction, including Hirschsprung disease, inflammatory bowel disease, irritable bowel syndrome, and colorectal cancer^2,3^. To study the role of the ENS in these diseases, several mouse models with altered ENS composition have been developed. While mouse models presenting with aganglionosis (e.g. knockout of Edn3, Ret, Col6a4 or Phox2B)^4^ or a reduced number of enteric neural cells (e.g. knockout of ErbB3 or loss of Hand2)^5,6^ are widely used, few mouse lines with an increase density of enteric neurons are available and each with their own limitations. The Gas1^tm2Fan/tm2Fan^ mouse model has increased enteric neuron numbers but is lethal at birth in its homozygous form^7^, while the Kif26a^tm1.1Noh/tm1.1Noh^ mouse model has enteric neuronal hyperplasia and megacolon, but is lethal around P15^8^. The Tyr:Cre/°;PTENF/F mouse model also presents with enteric neuronal hyperplasia and hypertrophy, but is lethal between P13-20^9^. The Tg.(Myog-Gdnf)1Lich model only has increased numbers of submucosal neurons^10^. The *neuron specific enolase*(*NSE)-Noggin* model (Tg.(Eno2-Nog-EGFP)Alch) is, to our knowledge, the only viable mouse model with increased neuron numbers in the submucosal and myenteric plexus described so far^11,12^, and is therefore most suitable for studies in adult mice that require increased neuronal density in the entire ENS.

The *NSE-Noggin* mice were generated by pronuclear injection of an *NSE-Noggin* construct, created by subcloning *Noggin* into a bicistronic internal ribosomal entry site (IRES) vector containing enhanced green fluorescent protein (*EGFP*) for ease of transcript detection, and subsequent subcloning downstream of the rat *NSE* promoter. This results in an overexpression of Noggin and expression of EGFP in all cells expressing NSE, notably neurons. Noggin is a secreted glycoprotein that antagonizes bone morphogenetic protein (BMP) signaling, which is critical for ENS development^12,13^. Altering the expression of noggin therefore affects ENS development, but a conditional model is required to prevent affecting gut morphogenesis during the earliest stages of embryogenesis^14^. The *NSE* promotor is only expressed in differentiated neurons^15^ and becomes significant at E15-16^11^, when colonization of the gut by enteric neural crest-derived cells is completed, which makes it suitable to study the effects of Noggin on the ENS. The *NSE-Noggin* transgenic mouse model was generated and first described by Guha et al.^16^, proving the expression of *Noggin* transgene by neuronal cells. The transgene was also mis-expressed by hair matrix cells, leading to the use of this model for studying hair follicle differentiation.

*NSE-Noggin* mice are described to have a higher density of enteric neurons due to the antagonism of Noggin on BMP signaling, which normally limits the expansion of the ENS^11,12^. More specifically, *NSE-Noggin* transgenic mice are reported to develop less CGRP-positive and more serotonin, calbindin, and calretinin-positive neurons^11^. As a consequence of their altered ENS composition, *NSE-Noggin* mice have an increased faecal output frequency, higher stool water content, and irregular gastrointestinal transit. *NSE-Noggin* mice have been used as a model for gut hyperinnervation to study the effect of enteric neuronal density on inflammation^17^. With the goal to investigate the effects of an increased enteric neuronal density on colorectal cancer, we aimed to validate the *NSE-Noggin* mouse model and to replicate the findings previously reported^11,17^.

## Methods

### Mice

*NSE-Noggin* transgenic mice were kindly provided by Dr. K.G. Margolis from Columbia University, New York, with permission from the creator of the mouse line Dr. J.A. Kessler from Northwestern University, Chicago. Due to the presence of pathogens (Murine Norovirus (MNV), *Helicobacter ganmani*, *Helicobacter typhlonius*, and *Pasteurella pneumotropica*), embryos were generated, cryopreserved and later rederived by embryo transfer in specific pathogen-free (SPF) Swiss foster mice to generate SPF *NSE-Noggin* mice. Animals were housed in an SPF breeding facility and health monitoring was performed according to FELASA 2014 guidelines (Supplementary Table S1). Hemizygous *NSE-Noggin* mice were intercrossed to generate wild-type (WT) and homozygous (HO) *NSE-Noggin* offspring of both sexes for experiments. No backcrossing or outcrossing to other strains has been performed after rederivation. Animals were preferably co-housed in groups of maximum 5 animals per individually ventilated cage with ad libitum access to water and food. All animal experiments were conducted with approval from the Committee of Animal Welfare of Maastricht University and according to Dutch regulations (AVD1070020174386), and methods were reported in accordance with ARRIVE guidelines.

### Genotyping

All mice were characterized by genotyping quantitative polymerase chain reaction (qPCR) on DNA and visual observation of the fur to determine their genotype before use in experiments. DNA was extracted from toes by incubating them in 50 mM KOH at 95 °C for 1 h. The qPCR reaction mix contained 6 µl SYBR™ green PCR Master Mix (Bio-Rad), 1 µl primer mix (10 µM; IDT), and the extracted DNA in a final volume of 12 µl. Primer sequences were EGFP-Forward 5′-ACCACTACCTGAGCACCCAGTC-3′ and EGFP-Reverse ′5-GTCCATGCCGAGAGTGATCC-3′ for the transgene, and GAPDH-Forward 5′-CAACTCACTCAAGATTGTCAGCAA-3′, and GAPDH-Reverse 5′-TGGCAGTGATGGCATGGA-3′ as a control to normalize DNA quantity. The qPCR was performed using the Bio-Rad CFX96™ Real-Time PCR system with the following conditions: (I) initial denaturation: 95 °C for 10 min, (II) 40 cycles: 95 °C for 30 s (denaturation), 57 °C (annealing) for 30 s, 60 °C (extension and plate reading) for 30 s, and (III) final melt curve analysis going from 65 to 95 °C with 0.5 °C increment.

For gel electrophoresis, a regular PCR was performed with a PCR reaction mix containing 10 μl REDExtract-N-Amp PCR Reaction Mix (Sigma-Aldrich), 1.6 μl primer mix (10 μM), and the extracted DNA in a final volume of 20 μl. The EGFP-Forward and EGFP-Reverse primers were used in combination with positive internal control primers: Ctrl-Forward 5′-CAGCGCCGCAACTATAAGAG-3′ and Ctrl-Reverse 5′-CATCGACCGGTAATGCAG-3′. The PCR was performed on a Bio-Rad T100™ thermal cycler (Bio-Rad) with initial incubation at 95 °C for 10 min, then 35 cycles 95 °C for 10 s, 57 °C for 10 s, and 72 °C for 30 s, and finally elongation at 72 °C for 4 min. PCR product was detected in a 1.5% (w/v) agarose gel in 0.5 × Tris–borate-EDTA (TBE) buffer. The transgene can be detected as a 110 bp fragment, while the control fragment can be detected by a 200 bp band.

### DNA sequencing

The presence of the NSE-Noggin-IRES-EGFP construct in transgenic mice was verified by sequencing. The target area was amplified using a nested PCR approach. A 1938bp segment (*NSE-EGFP*) was amplified, subsequently diluted and five smaller overlapping products were amplified (Table 1). After PCR, DNA sequencing was performed using BigDye® Terminator v1.1 Cycle Sequencing Kit (Thermo Fisher Scientific) and ABI 3730 DNA analyzer Kit (Thermo Fisher Scientific).

**Table 1:** List of primers used to obtain the DNA fragments of the NSE-Noggin-IRES-EGFP construct for subsequent DNA sequencing.

Primer name	Primer sequence	Reaction
**Outside PCR (1938 bp)**		
NSE FW	CCCCTAGGGACTGGAGACC	60 °C 35×
EGFP RV	CTCCTCGCCCTTGCTCA	
**Inside PCRs**		
PCR1 FW	CCCCTAGGGACTGGAGACC	60 °C 35×
PCR1 RV	GTGGACAAGAGGGAAGGAGAC	
PCR2 FW	GTCTCCTTCCCTCTTGTCCAC	60 °C 35×
PCR2 RV	GCGAAGTAGCCATAAAGCCC	
PCR3 FW	GGGCTTTATGGCTACTTCGC	60 °C 35×
PCR3 RV	ACACTCGGAAATGATGGGGT	
PCR4 FW	TCCCATCCAGTACCCCATCA	60 °C 35×
PCR4 RV	CAAACGCACACCGGCCT	
PCR5 FW	AGGCCGGTGTGCGTTTG	60 °C 35×
PCR5 RV	CTCCTCGCCCTTGCTCA	

### Gene expression

RNA was isolated from small pieces of adult proximal colon using the RNeasy Mini Kit (Qiagen) according to manufacturer’s instructions. Frozen tissues were lysed in 350 µl RLT buffer containing 1% β-mercaptoethanol using a pellet pestle and DNase I solution was used to remove DNA. Isolated RNA was dissolved in 60 µl RNA-free water and stored at − 80 °C until further analysis. Reverse transcription was carried out with the iScript™ Reverse Transcription kit (Bio-Rad). Subsequently, qPCR was performed using SYBR™ green PCR Master Mix (Bio-Rad) on the Bio-Rad CFX96™ Real-Time PCR system. Noggin expression was quantified using two different primer sets: (1) Noggin-Forward 5’-GAGGACCTGCGGAGCT-3′ and Noggin-Reverse ′5-ACAGCGCCACCGCAGCA-3′ (2) Noggin-Forward 5′-CCTGGTGGACCTCATCGAAC-3′ and Noggin-Reverse ‘5-GGGGGCGAAGTAGCCATAAA-3′. Values were normalized using the primers PGK1-Forward 5′-GAAGGGAAGGGAAAAGATGC-3′ and PGK1-Reverse 5′- GCTATGGGCTCGGTGTGC -3′.

### Sample preparation

Mice were sacrificed using CO_2_/O_2_ asphyxiation at the age of 4 weeks (N = 4 WT, 3 males, 1 female; N = 5 HO, 4 males, 1 female) and 10 weeks (N = 7 WT, 4 males, 3 females; N = 7 HO, 5 males, 2 females), the abdomen was cut open and the gastrointestinal tract was removed from stomach to anus. Colons were cut open along the mesentery and further dissected as previously described^18^. In brief, the colon was pinned flat in a Sylgard®-coated Petri dish containing PBS and the mucosal and submucosal layers were removed using forceps. The remaining myenteric plexus preparations were fixated in 4% paraformaldehyde (PFA) for 30 min, washed with PBS and stored at 4 °C until further processing. Fixated preparations were permeabilized and blocked with 4% donkey serum (Jackson ImmunoResearch) in 1% Triton X-100 in PBS for 2 h at RT and subsequently incubated with primary antibodies (Table 2) for 16–24 h at 4 °C, washed, and incubated with secondary antibodies (Table 2) for 2 h at 4 °C. Tissues were washed and mounted on silane-coated glass slides (VWR) using Citifluor with or without DAPI (Aurion).

**Table 2:** List of antibodies used for immunofluorescence.

Antibody	Host	Dilution	Supplier	Cat. no.
**Primary antibodies**				
GFP	Rabbit	1:200	Abcam	ab290
Hu	Human	1:20,000	Gift from Vanda Lennon	
Calbindin	Rabbit	1:20,000	Swant	CB-38a
Calretinin	Goat	1:500	Swant	CG1
Serotonin	Goat	1:1000	Abcam	ab66047
**Secondary antibodies**				
Anti-human Alexa 594	Goat	1:1000	Thermo Fisher	A-11014
Anti-human Dylight 650	Donkey	1:1000	Thermo Fisher	SA5-10129
Anti-rabbit Alexa 488	Donkey	1:1000	Thermo Fisher	A-21206
Anti-goat Alexa 594	Donkey	1:1000	Thermo Fisher	A-11058

### Microscopic imaging and analysis

Presence of Noggin-EGFP protein in distal colon preparations of *NSE-Noggin* HO mice and absence in WT mice was visualized with an Olympus BX63 automatic fluorescence microscope (FLIR Machine Vision camera; 60×, oil immersion lens), and analyzed using BioView Duet 3.7.2.3 and ImageJ 1.52p (NIH). Hu, calretinin, calbindin, and serotonin-positive neurons were counted by a blinded observer in the muscle/myenteric plexus preparations of the proximal and distal colon of *NSE-Noggin* WT and HO mice to assess neuronal density. A Leica TCS SP8 confocal microscope (Fluotar VISIR; 25×, H_2_O immersion lens, NA = 0.95) was used to make tilescans of approximately 2.0 mm^2^ per region and at least two regions per tissue were imaged. Hu, calretinin, calbindin, and serotonin-positive cells were manually counted using the Cell Counter plugin in ImageJ 1.52p (NIH). The cell number per region was averaged per preparation per mouse and normalized for colon stretching by multiplying myenteric neuron number with a factor calculated as total stretched colon length after dissection divided by total colon length before dissection. Tissues that were damaged were excluded from analysis, which only occurred in calretinin subtype analysis (excluded: 1 WT for proximal colon and 1 WT vs 2 HO in distal colon).

### Statistical analysis

Data were analyzed by a Student’s t-test for the comparison of *NSE-Noggin* HO with *NSE-Noggin* WT using GraphPad Prism version 5. Mean, standard deviation, and *P*-value are reported, mean and standard error of mean (SEM) are visualized in the graphs.

## Results

### NSE-Noggin mice can be genotyped based on transgene presence and based on fur

**Figure 1:**
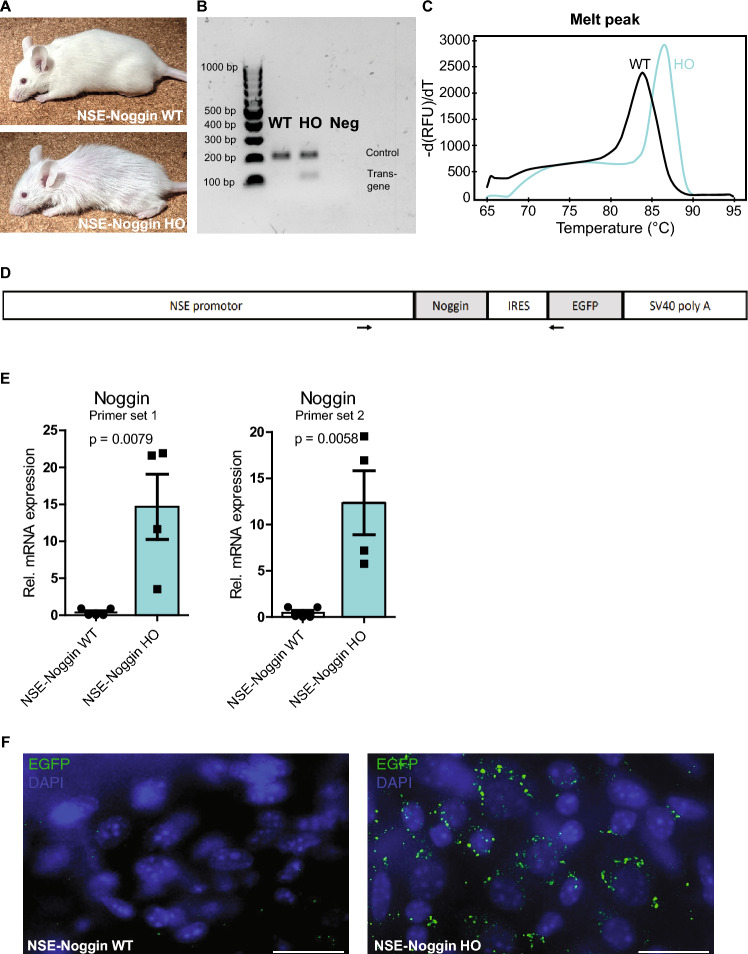
Phenotyping, genotyping and genetic characterization of the NSE-Noggin model. (**A**) *NSE-Noggin* HO mice have dramatic hair loss compared to *NSE-Noggin* WT mice and can be distinguished based on phenotype. (**B**) Genotyping PCR and gel electrophoresis show the presence of the transgene at 110 bp and the internal positive control for presence of DNA at 200 bp. Neg = negative control. (**C**) Genotyping qPCR results in different melt peaks for transgenic and WT mice. (**D**) The NSE-Noggin-IRES-EGFP construct is present in the *NSE-Noggin* HO mice based on DNA sequencing of a 1938 bp sequence indicated by arrows. The construct consists of 4 kb of rat NSE gene DNA with 2.8 kb 5′ flanking DNA, exon 1 (50 bp), intron 1 (1.2 kb), and 6 bp of exon 2, followed by the CDS of Noggin, IRES, EGFP and SV40 polyadenylation signal. (**E**) *Noggin* is significantly higher expressed in *NSE-Noggin* HO mice compared to *NSE-Noggin* WT mice confirmed by quantitative PCR analysis with two different primer sets. (**F**) Immunoreactivity for the EGFP reporter is present in the myenteric plexus of *NSE-Noggin* HO mice and absent from *NSE-Noggin* WT mice, confirming translation of the Noggin-EGFP construct. Scale bar equals 20 µm.

Intercrossing of *NSE-Noggin* hemizygous (HEMI) mice resulted in offspring at the expected Mendelian frequency of 24.7% (116/469) *NSE-Noggin* WT, 50.3% (236/469) *NSE-Noggin* HEMI, and 24.9% (117/469) transgene-expressing *NSE-Noggin* HO mice. *NSE-Noggin* WT and *NSE-Noggin* HO mice could be readily distinguished by their fur (Fig. 1A), as Noggin-overexpressing mice present with substantial hair loss postnatally. In addition, genotyping using regular PCR and gel electrophoresis (Fig. 1B) as well as qPCR (Fig. 1C) showed a clear difference between WT and HO mice. The NSE-Noggin-IRES-EGFP DNA construct was validated by sequencing of a 1938 bp sequence encompassing the end of the *NSE promoter*, *Noggin*, *IRES* and the beginning of *EGFP* (Fig. 1D) and showed the presence of the complete construct in *NSE-Noggin* HO mice. Confirming transgene overexpression in the colon, *Noggin* expression levels were significantly increased in transgenic mice compared to WT using two different primer sets (*P* = 0.0079 and *P* = 0.0058, respectively; Fig. 1E). Furthermore, myenteric plexus preparations of *NSE-Noggin* mice showed the expression of the EGFP reporter in transgenic mice, verifying translation of the *Noggin* gene (Fig. 1F).

### NSE-Noggin mice do not have a higher neuronal density in the myenteric plexus

**Figure 2:**
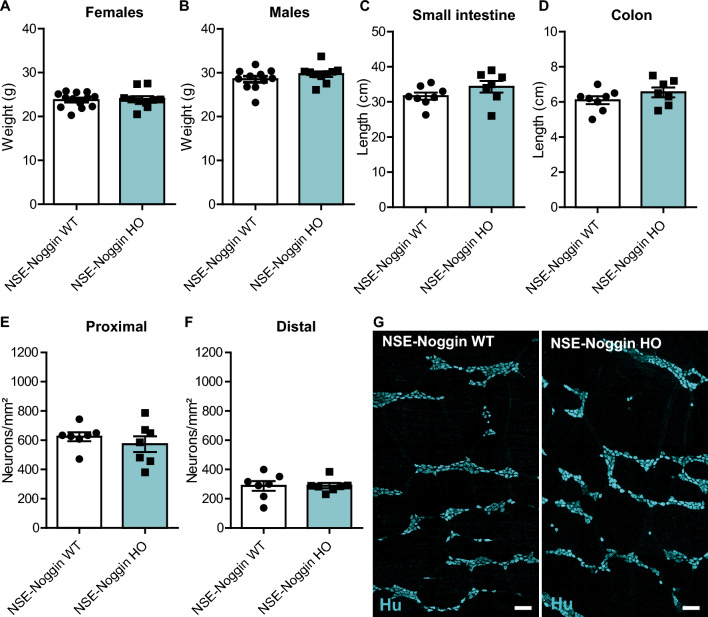
Characterization of the phenotype of *NSE-Noggin* mice. (**A**) Body weight is similar in *NSE-Noggin* WT and HO females (N = 13 vs N = 10) and (**B**) in males (N = 11 vs N = 10) at 10 weeks of age. (**C**) The length of the colon (N = 8 vs N = 7) and (**D**) small intestine (N = 8 vs N = 7) is also similar. (**E**) Enteric neuron number in proximal (N = 7 vs N = 7) and (**F**) distal (N = 7 vs N = 7) colon is the same, as shown by (**G**) immunofluorescent staining for Hu on colon myenteric plexus preparations of 10-week-old mice. Scale bar equals 100 µm.

Initially, we used 10-week-old mice (as this age was required for the originally-planned colorectal cancer induction studies). WT and HO mice had similar body weight (females 23.8 ± 1.7 g and 24.0 ± 2.1 g, *P* = 0.906; males 28.5 ± 2.3 g and 29.7 ± 2.0 g, *P* = 0.245; Fig. 2A, B), and small intestine (31.6 ± 2.8 cm and 34.3 ± 4.4 cm, *P* = 0.182; Fig. 2C) and colon (6.1 ± 0.6 cm and 6.5 ± 0.7 cm, *P* = 0.226; Fig. 2D) length. To assess neuronal density, colon myenteric plexus preparations were immunofluorescently stained for the pan-neuronal marker Hu, and manually counted while blinded for genotype. Even though *NSE-Noggin* mice express the transgene (Fig. 1), the myenteric neuron number in the colon was not different in *NSE-Noggin* WT and HO animals (proximal 624 ± 80 neurons/mm^2^ and 573 ± 141 neurons/mm^2^, *P* = 0.424; distal 288 ± 87 neurons/mm^2^ and 289 ± 47 neurons/mm^2^, *P* = 0.960; Fig. 2E–G), in contrast to what has been reported previously^11^.

As enteric neuronal density in the study by Chalazonitis et al.^11^ was assessed in younger mice, specifically 4 weeks old, we also quantified Hu-positive cells in 4-week-old *NSE-Noggin* mice to investigate if the conflicting results were due to age. However, again we found that the enteric neuronal density was similar in the colon myenteric plexus of *NSE-Noggin* HO and *NSE-Noggin* WT mice (proximal 898 ± 104 neurons/mm^2^ and 912 ± 116 neurons/mm^2^, *P* = 0.859; distal 355 ± 85 neurons/mm^2^ and 354 ± 47 neurons/mm^2^, *P* = 0.990; Supplementary Fig. S1E–G), as was their body weight and gut length (bodyweight females 18.3 ± 2.3 g and 17.8 ± 0.5, *P* = 0.222; bodyweight males 19.8 ± 2.0 g, P = 19.6 ± 3.1 g, *P* = 0.961; small intestine length 30.8 ± 3.5 cm and 29.3 ± 2.9 cm, *P* = 0.4558; colon length 6.5 ± 0.5 cm and 6.1 ± 0.5 cm, *P* = 0.203; Supplementary Fig. S1A–D).

### Calbindin, calretinin, and serotonin-positive neuron proportions are not affected in NSE-Noggin mice

**Figure 3:**
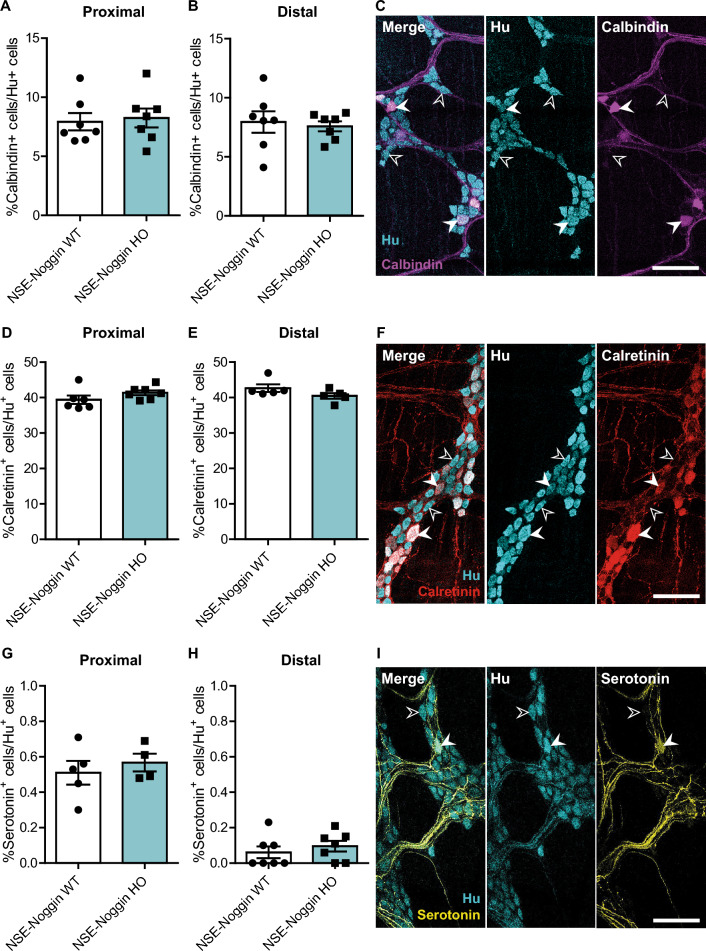
Neuronal subtype characterization as a proportion of the total amount of neurons. (**A**) *NSE-Noggin* WT and HO mice have an equal percentage of calbindin-positive enteric neurons in proximal (N = 7 vs N = 7) and (**B**) distal (N = 7 vs N = 7) colon, assessed by (**C**) immunofluorescent labeling for calbindin (magenta) and Hu (cyan). (**D**) The percentage of calretinin-positive enteric neurons is similar in proximal (N = 6 vs N = 7) and (**E**) distal (N = 6 vs N = 5) colon, assessed by (**F**) immunofluorescent labeling for calretinin (red) and Hu (cyan). (**G**) *NSE-Noggin* WT and HO mice have a similar percentage of serotonin-positive enteric neurons in proximal (N = 5 vs N = 4) and (**H**) distal (N = 7 vs N = 7) colon, assessed by (**I**) immunofluorescent labeling for serotonin (yellow) and Hu (cyan). All stainings were performed on colon myenteric plexus preparations of 10-week-old mice. Open arrows point to marker-negative Hu-positive cells, closed arrows point to calbindin, calretinin, or serotonin-positive enteric neurons. Scale bar equals 100 µm.

To investigate whether specific neuronal subtype proportions were altered, we employed immunofluorescent labeling in colons of 10-week-old animals to count calbindin, calretinin, and serotonin-positive neurons, which have been reported to be affected in the *NSE-Noggin* mouse model^11^. Calbindin, calretinin, and serotonin-positive cell numbers were normalized to the total enteric neuronal density by calculating the percentage of neurochemical marker-positive cells relative to Hu-positive cells. However, no alteration in the proportion of calbindin (proximal 7.9 ± 1.9% and 8.2 ± 0.8%, *P* = 0.770; distal 8.0 ± 2.4% and 7.6 ± 1.1%, *P* = 0.719; Fig. 3A–C), calretinin (proximal 39.4 ± 3.0% and 41.4 ± 1.8%, *P* = 0.166; distal 42.6 ± 2.4% and 40.5 ± 1.8%, *P* = 0.143; Fig. 3D–F), or serotonin-positive (proximal 0.51 ± 0.15% and 0.57 ± 0.10%, *P* = 0.535; distal 0.06 ± 0.09% and 0.10 ± 0.08%, *P* = 0.459; Fig. 3G–I) neurons was observed in the myenteric plexus of our *NSE-Noggin* HO compared to *NSE-Noggin* WT mice.

## Discussion

Mouse models are currently still necessary and useful tools for biomedical research. However, standardization of animal experiments has its limits, with many sources of phenotypic variability leading to reproducibility problems. This study could not replicate the outcome of an earlier study with the same *NSE-Noggin* transgenic mouse line. Whereas the construct, transgene expression and EGFP reporter expression could be validated, the mice did not present with the phenotypic characteristic of enteric hyperinnervation in the colon.

It is a well-known problem that animal studies often fail to be reproduced. It has been estimated that over 50% of preclinical studies, i.e. animals studies, are irreproducible^19^ due to weaknesses in study design, such as lack of blinding and randomization, or because of equipment or procedural differences between laboratories^20^. While examining the same outcome parameter of enteric neuron density, several differences exist between our experimental context and that of the previously reported findings using the same transgenic mouse line. First of all, it is performed in a different institute (Maastricht University, The Netherlands vs. Columbia University, New York^11^), which inevitably implies that factors related to animal housing and handling will be different, many of which might be unknown, unreported, or seemingly unimportant. Furthermore, the method to quantify the number of neurons is slightly different. The study by Chalazonitis et al.^11^ used Cuprolinic Blue as a neuronal counterstaining^21^, while this study employed an immunofluorescent labeling strategy with the pan-neuronal marker Hu. However, both methods have been compared in previous studies and are considered to give reliable and similar neuron counts^22,23^, and therefore are unlikely to cause the discrepancy in outcome. Furthermore, the group size for the neuronal quantifications in our study is based on power calculations using the original data of Chalazonitis et al.^11^ and required a sample size of 4 mice per group to reach statistical significance (alpha: 0.05, power: 80%), which we enlarged to 7 mice to increase the power and reliability of our data.

Genetic drift or other genetic alterations that occurred in the period between the study by Chalazonitis et al.^11^ which was published in 2008 and our study conducted in 2020–2023 could be a possible cause for differences observed between both studies. However, in our study, we showed the presence of the NSE-Noggin-IRES-EGFP construct, the increased expression of *Noggin* in the colon of adult transgenic mice and the translation of the *Noggin* gene, rejecting a role for genetic and transcriptional changes as cause of the altered phenotype.

Voelkl and Würbel state that there is inherent biological variation in mice, that is largely caused by environmental variation^24^. Countries, institutes, and even laboratories within the same institute can differ in a plethora of environmental factors. Diet, light conditions, temperature, housing conditions, animal handling, and many other factors can impact research outcomes^25–29^. For instance, a recent study showed that the variation caused by executing an experiment in different laboratories was larger than the effect of different experimenters^30^, confirming earlier findings that the variation between laboratories is a major source of study variability^31^.

An important environmental factor that can impact the mouse phenotype, the microbiome, has emerged as a variable as well^32^. Already in 2008, Ivanov et al.^33^ observed a difference in the number of Th17 cells in the small intestine of C57Bl/6 mice from different vendors. Mice from the Jackson Laboratory had lower numbers compared to mice from other sources, which was attributed to the lack or presence of specific bacterial species. Introducing these bacterial species through fecal transplantation reversed the phenotype in germ-free mice, implicating an important and causal role for intestinal microbiota.

For the ENS, the microbiome is of utter importance as it regulates ENS development and homeostasis^34^. For example, butyrate, a short chain fatty acid produced by microbiota, modulates the postnatal development of the myenteric glial network^35^, and germ-free mice have less neurons per ganglion and less nerve fibers postnatally^36^. While another study reported that the number of myenteric neurons was unchanged in germ-free mice, they observed a decrease in mucosal innervation^37^. Interestingly, colonization of germ-free mice with conventional microbiota rescued this phenotype, likely via activation of 5-HT_4_ receptors. Obata et al.^38^ discovered a transcription factor, aryl hydrocarbon receptor (AHR), that acts as a biosensor to link the microbial environment to ENS function. Relevant to our findings and linking directly to the inhibitory effect of Noggin on the BMP pathway, *Helicobacter pylori* was found to lead to an increase in BMP inhibitors, which could contribute to the hyperinnervation phenotype^39^.

In our study, the pathogens Murine Norovirus (MNV), *Helicobacter ganmani*, *Helicobacter typhlonius*, and *Pasteurella pneumotropica* have been eradicated from the *NSE-Noggin* microbiome by rederiving cryopreserved embryos in SPF mice in 2020. MNV, *Helicobacter ganmani* and *Helicobacter typhlonius* can induce intestinal inflammation and affect immune function^40–43^. In keeping with the importance of microbiota for ENS structure and function^2,34–38,44^, and the fact that the microbiome of the *NSE-Noggin* mice has changed upon introduction to the animal facility of Maastricht University (NL), altered microbiota composition should be considered as a likely cause for the contrasting results observed in our study and that of Chalazonitis et al.^11^. A list of microorganisms absent from the *NSE-Noggin* breeding colony in our institute is presented in Supplementary Table S1. However, we cannot make a direct comparison between the pathogenic load of the mice used for the study of Chalazonitis et al.^11^ and our study, as pathogen analysis of the mice used for the original study published in 2008 is not available. Notwithstanding the possible role of numerous other factors, comparisons of ENS-related outcomes between studies using animal models in which the microbiome is different should be performed carefully.

In conclusion, our findings indicate that for any study relying on previously established animal models, it is crucial to validate the reported phenotype. Furthermore, issues with reproducibility should stimulate the scientific community to investigate in detail the influence of the microbiota and other environmental factors on their models, and how these affect experimental results.

### Supplementary Information

Supplementary Information.

## Data Availability

The data supporting the findings of this study are available within the paper and its Supplementary Information. The raw sequence data reported in this paper have been deposited in the Genome Sequence Archive (Genomics, Proteomics & Bioinformatics 2021) in National Genomics Data Center (Nucleic Acids Res 2022), China National Center for Bioinformation/Beijing Institute of Genomics, Chinese Academy of Sciences (GSA: CRA014041) that are publicly accessible at https://ngdc.cncb.ac.cn/gsa.
