# Supplementary material for: Differences in enteric neuronal density in the NSE-Noggin mouse model across institutes
Source: Sci Rep. 2024 Feb 14;14:3686. doi: 10.1038/s41598-024-54337-w (PMC10866904; doi:10.1038/s41598-024-54337-w)
Supplement: Supplementary file 1 — Supplementary Information. [file 41598_2024_54337_MOESM1_ESM.pdf]

Supplementary Information

Differences in enteric neuronal density in the NSE-Noggin mouse model across institutes

Simone L. Schonkeren[1]*, Meike S. Thijssen[1,2]*, Musa Idris[1,3], Kim Wouters[1], Joëlle de Vaan[1], Andreas Teubner[4], Marion J. Gijbels[1,5], Werend Boesmans[1,2], Veerle Melotte[1,3]

Supplementary Table S1

Supplementary Table S1| List of Microorganisms absent from the *NSE-Noggin* breeding colony at Maastricht University. Results are based on screening of direct contact sentinels and colony animals according to FELASA 2014 guidelines for health monitoring of rodent colonies.

**Viruses**	*Adenovirus FL, Adenovirus K87, Ectromelia virus, General parvovirus (rNS-1), Lymphocytic choriomeningitisvirus, Minute virus of mice, Mouse hepatitis virus, Mouse parvovirus (rVP2), Mouse rotavirus / EDIM, Murine norovirus, Pneumonia virus of mice, Reovirus type 3, Sendai virus, Theiler's encephalomyelitis virus (GD VII)*
**Bacteria**	*Citrobacter rodentium, Corynebacterium kutscheri, Rodentibacter spp. (former P. pneumotropica), Salmonella spp., Streptobacillus moniliformis, Streptococci beta-haemolytic Group A, Streptococci beta-haemolytic Group B, Streptococci beta-haemolytic Group C, Streptococci beta-haemolytic Group G, Streptococcus pneumoniae, Helicobacter bilis, Helicobacter hepaticus, Helicobacter typhlonicus, Clostridium piliforme, Mycoplasma pulmonis*
**Parasites**	*Aspiculuris tetraptera, Chilomastix bettencourti, Eimeria spp., Giardia spp., Myobia musculi / Radfordia sp., Myocoptes musculinus, Other ectoparasites, Other endoparasites, Spironucleus muris, Syphacia obvelata, Trichomonas spp.*

Supplementary Figure S1

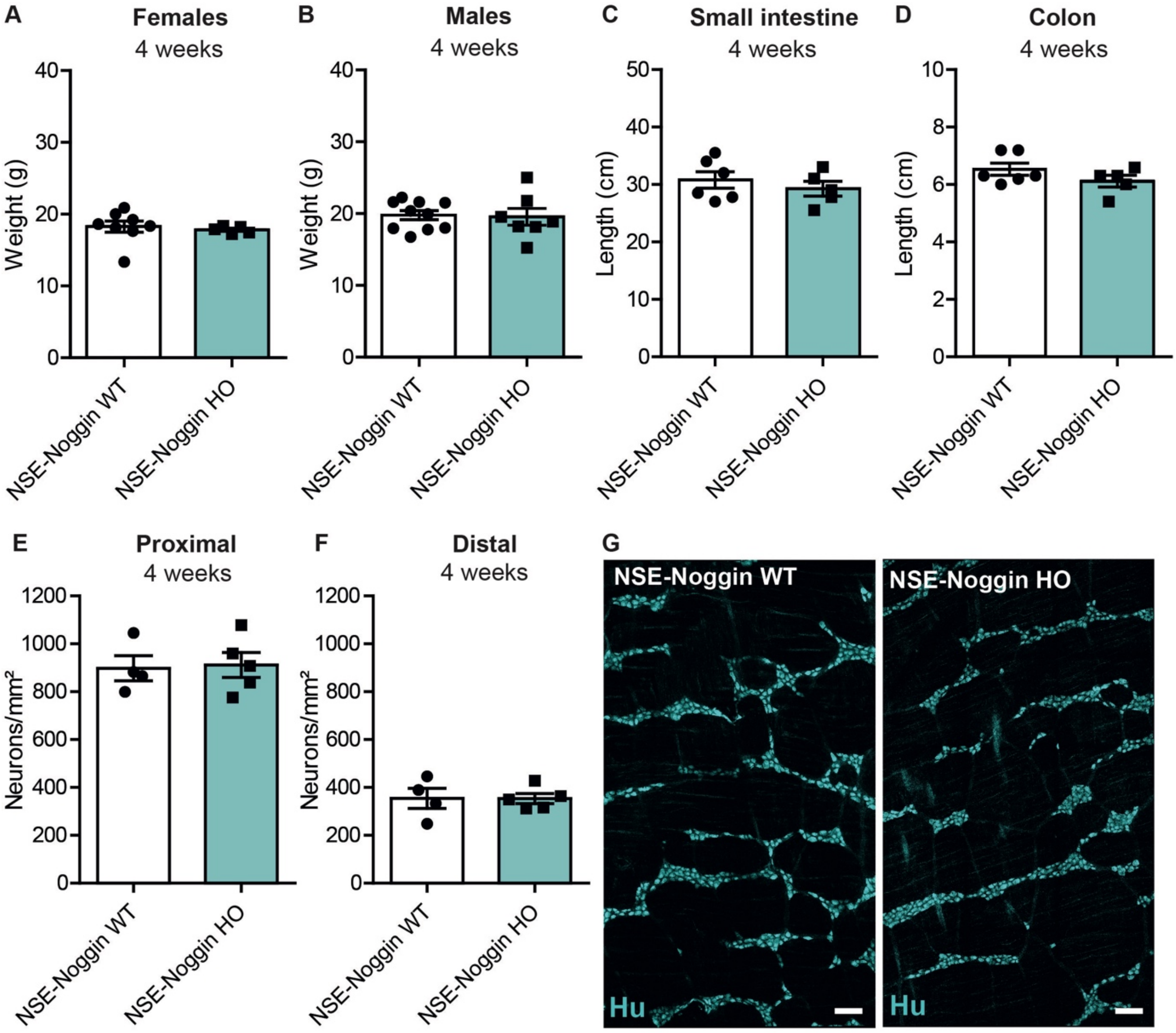

Supplementary Figure S1| Characterization of the phenotype of *NSE-Noggin* mice at the age of 4 weeks **A|** Body weight is similar between NSE-Noggin WT and HO in females (N=8 vs N=5) and **B|** in males (N=10 vs N=7) at 4 weeks of age. **C|** The length of the colon (N=6 vs N=5) and **D|** small intestine (N=6 vs N=5) is also similar. **E|** Enteric neuron number in proximal (N=4 vs N=5) and **F|** distal (N=4 vs N=5) colon is the same. **G|** Immunofluorescent staining for Hu on colon myenteric plexus preparations of 10-week-old mice. Scale bar equals 100 µm.
